# The treatment with pasireotide in Cushing’s disease: effect of long-term treatment on clinical picture and metabolic profile and management of adverse events in the experience of a single center

**DOI:** 10.1007/s40618-019-01077-8

**Published:** 2019-07-16

**Authors:** C. Simeoli, R. Ferrigno, M. C. De Martino, D. Iacuaniello, F. Papa, D. Angellotti, C. Pivonello, R. Patalano, M. Negri, A. Colao, R. Pivonello

**Affiliations:** Dipartimento di Medicina Clinica e Chirurgia, Sezione di Endocrinologia, Università “Federico II” di Napoli, Via Sergio Pansini 5, 80131 Naples, Italy

**Keywords:** Pasireotide, Cushing’s disease, Clinical picture, Metabolic syndrome, Management of adverse events

## Abstract

**Purposes:**

Pasireotide is the first medical therapy officially approved for adult patients with Cushing’s disease (CD) experiencing failure of pituitary surgery or not candidates for surgery. The current study aimed at investigating pasireotide effects on clinical picture and metabolic profile in patients enrolled in the phase III CSOM230B2305 trial at Naples center. In addition, the current study focused on safety issues encountered during the study, detailing the management of the different adverse events associated with the treatment with pasireotide in Naples center.

**Methods:**

Fourteen patients entered the study; eight patients, receiving pasireotide for at least 6 months, were considered for the efficacy analysis, whereas the entire cohort of 14 patients was considered for the safety analysis.

**Results:**

Full or partial disease control was obtained in 85.7% of patients, according to a “per-protocol” methodology analysis, and in 42.9% of patients, according to an “intention-to-treat” methodology analysis, after 12 months of treatment. A relevant improvement in clinical signs and symptoms, mainly in facial rubor, supraclavicular fat pad, bruising, hirsutism, and muscle strength was observed; body weight, body mass index, and waist circumference significantly reduced, and a slight non-significant reduction was observed in the prevalence of visceral obesity, hypercholesterolemia, and hypertriglyceridemia. Deterioration of glucose metabolism represented the most common adverse event, occurring in 71.4% of patients, and requiring a dietary regimen as first step, metformin therapy and/or long-acting insulin as second step, and short-acting insulin, as third step; no patients discontinued treatment for hyperglycaemia. Additional adverse events of interest were nausea (21.4%), and vomiting (14.3%), spontaneously resolved in few weeks or some months, except in one patient unsuccessfully treated with metoclopramide and ondansetron, and diarrhoea (14.3%), improved with loperamide treatment. Millimetric gallstones and biliary sludge (7.1%) were managed with ursodeoxycholic acid, inducing lithiasis and biliary sludge resolution, whereas hypocortisolism-related adverse events (7.1%) were resolved with a reduction in the pasireotide dose.

**Conclusions:**

The current study on a limited series of patients contributes to confirm that pasireotide may be considered a valid option for treatment of patients with CD, although it requires an appropriate management of adverse events, especially hyperglycaemia.

## Introduction

Cushing’s disease (CD), or pituitary-dependent Cushing’s syndrome (CS), is caused by an adrenocorticotrophic hormone (ACTH)-secreting pituitary tumor, leading to chronic hypercortisolism, which results in a typical clinical picture; this is characterized by weight gain with moon face, facial plethora, buffalo hump, supraclavicular and dorsal fat pads, cutaneous purplish striae, bruising, proximal myopathy, and hirsutism and/or acne [1–5]. The clinical picture is complicated by several comorbidities, including metabolic syndrome, characterized by visceral obesity, systemic arterial hypertension, impairment of glucose metabolism and dyslipidaemia, strictly associated with cardiovascular diseases, and additional clinical complications such as musculoskeletal diseases, impairment of reproductive and sexual function, neuropsychiatric diseases and immune disorders with higher susceptibility to infections [2]. The constellation of these comorbidities is associated with an increased morbidity and mortality, mainly for cardiovascular events and generalized sepsis, and with an impairment of quality of life (QoL) [1–5].

The aims of CD treatment include the normalization of cortisol secretion, the reversal of symptoms and signs of hypercortisolism, the removal of the tumor mass or control of tumor growth, with preservation of pituitary function, and the induction of a long-term disease control without recurrence [1, 6, 7].

Pituitary surgery is generally considered the first-line treatment of CD, but it is associated with unsuccessful outcome in an average of 25–50% and up to 70% of cases, due to immediate persistence or late recurrence of the disease [1, 6, 7]. Therefore, additional second-line treatments are frequently required, such as repeat pituitary surgery, pituitary radiotherapy, bilateral adrenalectomy, and medical therapy [1, 6, 7].

Pasireotide, a multireceptor-targeting somatostatin analogue (SSA), which targets the ACTH-secreting pituitary tumor, is the first medical therapy approved for the treatment of adult CD patients for whom surgery has failed or is not an option [1, 7]. Its high affinity for the somatostatin receptor subtype 5 (SSTR5), abundantly expressed in ACTH-secreting pituitary tumors, results in ACTH and consequently cortisol inhibition [1, 6, 7]. Although the genetic basis underlying the development of CD and the prediction of treatment susceptibility remain unclear, recently, a role of ubiquitin-specific protease 8 (USP8), a protein with deubiquitinase (DUB) activity that modulates the lysosomal turnover of several growth factor receptors, has been suggested [8]. A recent study demonstrated higher SSTR5 expression levels in USP8 mutant tumors, suggesting that the presence of USP8 mutations may predict favourable responses to pasireotide treatment [9].

The phase III clinical trial CSOM230B2305 investigated the efficacy and safety of the twice-daily subcutaneous (sc) pasireotide treatment, at the doses of 600 μg and 900 μg *bis in die* (bid), on the largest series of 162 CD patients [10]. Regardless of dose increase, full or partial disease control was obtained in 34.2% (600 μg bid) and 41.3% (900 μg bid) of patients after 6 months, and in 29.3% (600 μg) and 27.5% (900 μg) of patients after 12 months [10]. Pasireotide induced a significant improvement in clinical picture, with a positive impact on visceral obesity, hypertension and lipid profile, as well as on depression and health-related QoL [10–12]. Pasireotide was also found to induce relevant tumor shrinkage, after 12 months of treatment, with a mean percentage reduction of 9.1% in the 600 μg group and of 43.8% in the 900 μg group [10]. A marked reduction in tumor mass, and occasionally a radiological disappearance of the tumor, was also confirmed in a small cohort of patients with de novo or persistent CD, representing a subgroup of patients participating at the phase III trial in Naples center [13]. The safety profile of pasireotide was similar to that of the first-generation SSAs, except for the increased frequency and degree of hyperglycemia [10–12].

To our knowledge, despite some suggestions have been provided, mostly based on the pathophysiological mechanisms of hyperglycemia induced by pasireotide [14, 15], the best way to manage hyperglycemia, according to clinical practice, is still a matter of debate. Moreover, few recommendations are available regarding the management of the different adverse events (AEs) associated with the treatment with pasireotide.

The current study, although on a limited series of patients, describes the efficacy, focusing on clinical picture and metabolic profile, and details the safety management of pasireotide treatment, reporting the experience of one of the major centers involved in the phase III trial.

## Methods

### Study design

The current study considered the group of patients enrolled in the phase III CSOM230B2305 clinical trial at “Federico II” University of Naples. The study was approved by the local ethics committee and complied with the Declaration of Helsinki, in line with the Guidelines for Good Clinical Practice. All patients provided written informed consent and confidentiality statement of data collection according to the Italian privacy policy.

### Patients

Sixteen adult CD patients were screened for the phase III clinical trial [10], but due to screening failures in two, 14 patients were definitively enrolled. Patient’s profile at study entry, which was detailed in a previous manuscript dedicated to the same study [13], is summarized in Table 1. Fourteen patients (12 women, two men, aged 39.07 ± 14.54 years), including 12 patients with microadenoma and two with macroadenoma, 10 patients with de novo and four with persistent CD, started treatment with pasireotide. However, six patients dropped out before reaching the 6-month follow-up due to lack of efficacy in one (16.7%) and spontaneous consent withdrawal in five (83.3%). All six patients who dropped out before reaching the 6-month follow-up were not considered for the definitive efficacy analysis. Therefore, eight patients (seven women and one man, aged 38.9 ± 17.6 years), including six patients with de novo and two with persistent CD, among which seven with microadenoma and one with macroadenoma, were considered for the definitive efficacy analysis (Table 2). The entire group of 14 patients who started pasireotide treatment was considered for the safety analysis.

**Table 1:** Profile of the 14 patients enrolled into the global study

Patients’ number	14
F/M	12/2
Patients’ age (years)	39.07 ± 14.54
Microadenoma nr. (%)	12 (85.7)
Macroadenoma nr. (%)	2 (14.3)
De novo/persistent CD	10/4
Median pasireotide starting dose (µg bid)	900

**Table 2:** Profile of the eight patients considered for the study efficacy analysis

Patients’ number	8
F/M	7/1
Patients’ age (years)	38.9 ± 17.6
Microadenoma nr. (%)	7 (87.5)
Macroadenoma nr. (%)	1 (12.5)
De novo/persistent CD	6/2
Median pasireotide starting dose (µg bid)	900

### Study protocol

The study protocol has been detailed in a previous manuscript dedicated to the same study [13]. The endpoints of the current study were the evaluation of pasireotide effects on clinical picture, in terms of changes in clinical signs and symptoms of hypercortisolism, as well as on biochemical, and metabolic parameters, at months 6 and 12 in the eight patients completing the study, and the detailed evaluation of the safety profile, with an overview on AEs management, in the entire group of 14 patients entering the study. All the efficacy evaluations were assessed in relation to the degree of urinary free cortisol (UFC) response.

### Assessment of efficacy and disease severity

On the basis of UFC change or normalization, patients were classified as fully controlled (FC) if the UFC levels were ≤ the upper limit of the normal range (ULN) (145 nmol/24 h; 52.7 μg/24 h), partially controlled (PC) if UFC levels were > ULN, but reduced by ≥ 50% from baseline, and uncontrolled (UC) if UFC levels were > ULN and reduced by < 50% from baseline. Disease severity was evaluated according to baseline UFC levels, and patients were classified to have mild (ULN ≤ 2), moderate (ULN > 2 and ≤ 5), severe (ULN > 5 and ≤ 10), and very severe (ULN > 10) hypercortisolism.

### Assessment of clinical picture

Facial rubor, supraclavicular and dorsal fat pads, bruising, cutaneous purplish striae, and hirsutism were evaluated by means of observation of photographs of patients throughout the study by a blinded reviewer, with experience in treating CD patients, but not involved in different aspects of the study; the reviewer had no direct contact with study patients and was unaware of both the study group assignments and the timing of the photographs, as per protocol [10]. Two photographs (one frontal and one dorsal from the shoulders to the vertex of the head) were used to assess facial rubor, and supraclavicular and dorsal fat pads [10]. Two additional photographs (one frontal and one dorsal of the trunk with the patient in a standing position) were used to assess bruising, striae, and hirsutism [10]. The severity of facial rubor, supraclavicular and dorsal fat pads, bruising and striae was scored on a scale severity level ranging from 0 to 3 (0 = no signs; 1 = mild; 2 = moderate; 3 = severe) [10]. Hirsutism was measured according to the Ferriman–Gallwey score (FGS): the extent of hair growth in nine locations was rated 0–4, resulting in a score of 0–36 [10]. Proximal muscle strength was assessed by placing patients in a low seated position, then having them extend their arms in front of them and stand up. Patients were evaluated using a specific scale (3 = completely unable to stand; 2 = able to stand using arms as assistance; 1 = able to stand after several efforts without using arms as assistance; 0 = able to stand easily with arms extended) [10].

### Assessment of metabolic parameters

Body weight (BW), body mass index (BMI), waist circumference (WC), systolic blood pressure (SBP), and diastolic blood pressure (DBP) were assessed at the baseline and, thereafter, at each timepoint during the study, as per protocol [10]. In particular, BW was measured using a calibrated balance, and height was recorded at screening; these measurements were used to calculate BMI. According to BMI, patients were classified in normal weight (BMI < 25 kg/m^2^), overweight (BMI ≥ 25 and < 30 kg/m^2,^) and obese (BMI ≥ 30 kg/m^2^). WC was measured midway between iliac crest and lowest rib margin in the left and right mid-axillary lines. Visceral obesity was defined as WC ≥ 94 cm in men and ≥ 80 cm in women, according to IDF criteria [16]. SBP and DBP were recorded as mean of three values at 1–2 min intervals in sitting posture after 5 min of rest, using a standard mercury sphygmomanometer. Systemic arterial hypertension was defined as SBP ≥ 140 mmHg and/or DBP ≥ 90 mmHg, according to guidelines of American Society of Hypertension and the International Society of Hypertension [17], or treatment of a previously diagnosed systemic arterial hypertension. Total cholesterol, low-density lipoprotein (LDL) cholesterol and high-density lipoprotein (HDL) cholesterol, triglycerides, fasting plasma glucose (FPG), and glycated haemoglobin (HbA1c) were performed by central laboratories. Lipid profile was evaluated according to ATP-III guidelines’ criteria [18]. Hypercholesterolemia was defined as total cholesterol ≥ 200 mg/dL (≥ 5.17 mmol/L) or LDL cholesterol ≥ 130 mg/dL (≥ 3.36 mmol/L) or HDL cholesterol < 40 mg/dL (< 1.03 mmol/L) in men and < 50 mg/dL (1.3 mmol/L) in women [18], or specific treatment for hypercholesterolemia. Hypertriglyceridemia was defined as triglycerides ≥ 150 mg/dL (≥ 1.7 mmol/L) [18] or specific treatment for hypertriglyceridemia. The diagnosis of impaired fasting glucose (IFG), impaired glucose tolerance (IGT), and diabetes was performed according to the latest American Diabetes Association guidelines [19]. A prediabetic status, including IFG and/or IGT, was defined by FPG between 100 and 125 mg/dL (5.6–6.9 mmol/L) or HbA1C of 5.7–6.4% whereas diabetes was defined as FPG ≥ 126 mg/dL (≥ 7 mmol/L) or HbA1C ≥ 6.5%, or a random plasma glucose ≥ 200 mg/dL (≥ 11.1 mmol/L) in case of concomitant presence of classic symptoms and/or signs of hyperglycemia or hyperglycemic crisis [19], or treatments of a previously diagnosed diabetes. Metabolic syndrome (MS) was assessed in line with IDF criteria: central obesity defined by WC ≥ 94 cm in men and ≥ 80 cm in women plus any two among the following criteria: (1) triglycerides ≥ 150 mg/dL (≥ 1.7 mmol/L) or specific treatment for hypertriglyceridemia; (2) HDL cholesterol < 40 mg/dL (< 1.03 mmol/L) in men and < 50 mg/dL (1.3 mmol/L) in women or specific treatment for hypercholesterolemia; (3) SBP ≥ 130 mmHg/DBP ≥ 85 mmHg or specific treatment of previously diagnosed systemic arterial hypertension; and (4) FPG ≥ 100 mg/dL (5.6 mmol/L) or previously diagnosed type 2 diabetes [16].

### Assessment of safety

AEs were graded, according to Common Terminology Criteria for adverse events (CT-CAE) version 3 [20], on the basis of severity in grade 1 (mild), 2 (moderate), 3 (severe), and 4 (very severe, life-threatening, or disabling). The interventions performed for the AEs management were also carefully registered during the entire period of the study in all 14 patients included.

### Statistical analysis

Data were analyzed using SPSS Software for Windows, version 20.0 (SPSS, Inc., Cary, NC package). Data are reported as mean ± standard deviation (M ± SD) or as percentages. The comparison between the numerical data before and after pasireotide treatment was performed by non-parametric Wilcoxon test. The comparison between the numerical data among FC, PC, and UC patients, was performed by non-parametric Kruskal–Wallis and/or Mann–Whitney *U* test. The comparison between prevalences was performed by *χ*^2^ test. Significance was set at 5%. The analysis of clinical picture, as well as biochemical and metabolic parameters was performed for those patients still enrolled in the study and who had evaluable measurements at both baseline and at specific timepoints considered for the study analysis (6 and 12 months). The evaluation of the AEs was performed for the totality of patients during the entire period of treatment.

## Results

### Baseline characteristics and UFC

The baseline characteristics of 14 patients recruited in the current study have been reported in a previous manuscript dedicated to the same study [13]. Six (42.85%) patients dropped out from the study before reaching the 6-month follow-up due to lack of efficacy in one (16.7%) case and spontaneous consent withdrawal in five (83.3%) cases. In particular, the consent was withdrawn by the patients for subjective difficulties in managing AEs in three (60%) cases, poor compliance to the treatment in one (20%) case, and patient decision to undergo pituitary surgery in one (20%) case.

The eight (57.15%) patients, who continued the study and reached at least 6-month follow-up, were considered for the current study. These eight patients have been randomized to receive pasireotide sc 600 µg bid (two patients, 25%) or 900 µg bid (six patients, 75%). At baseline, UFC levels were 771.7 ± 606.6 nmol/24 h (280.6 ± 220.6 µg/24 h). Five (62.5%) patients had moderate, two (25%) severe, and one (12.5%) very severe hypercortisolism. After the first 3 months of treatment, all patients met the criteria to continue receiving the same dose for the following 3 months. At 6-month follow-up, at a median dose of 900 µg bid (range 600–900 µg bid), UFC levels significantly decreased by 65% compared to baseline (6 months: 236.5 ± 164.3 nmol/24 h, 86 ± 59.7 µg/24 h; *p* = 0.008).

According to a “per-protocol” methodology analysis, which considered for the analysis exclusively the cohort of eight patients who completed 6 months of treatment, excluding patients who discontinued the treatment, 37.5% (three) were FC, 37.5% (three) were PC, and 25% (two) patients were UC. On the basis of these evidences, a full or partial control of the disease was observed in 75% of cases, corresponding to six of eight patients who reached 6-month follow-up.

According to an “intention-to-treat” methodology analysis, which considered for the analysis the entire cohort of 14 patients starting treatment, including patients who discontinued treatment, because considered as unresponsive, 21.4% (three) were FC, 21.4% (three) were PC, and 57.2% (eight) patients were UC. On the basis of these evidences, a full or partial control of the disease was observed in 42.8% of cases, corresponding to six of the 14 patients enrolled in the study and starting treatment.

However, evaluating the six patients who discontinued treatment, two (33.3%) patients, who dropped out from the study 45 days after starting treatment, were FC, one (16.7%) patient, who dropped out from the study 90 days after starting treatment, was UC, considering the last available evaluation, whereas in the three (50%) patients, who dropped out from the study within 15 days after starting treatment, intermediate UFC evaluations were not available, and control of disease remained unknown.

UFC variably decreased in all the three groups of patients. Indeed, in the three FC patients, a mean UFC reduction of 84.3% (74.2–91.6%) was observed, whereas a mean UFC reduction of 64.9% (55.8–82.1%) and 36.4% (33.3–39.5%) was observed in the three PC and in the two UC patients, respectively.

According to the results at 6-month follow-up, the three FC patients maintained the same dose (900 µg bid), whereas a dose increase from 600 to 900 µg bid was required in two PC patients and from 900 to 1200 µg bid in one of the two UC patients. The remaining PC patient treated with 900 µg bid was offered to have a dose increase to 1200 µg bid, but refused and remained at 900 µg bid. The remaining UC patient withdrew the consent for poor compliance and discontinued the treatment at 6-month follow-up.

In the seven patients, who continued the study, at 12-month follow-up at a median dose of 900 µg bid (range 900–1200 µg bid), UFC levels were 204.8 ± 113.9 nmol/24 h (74.5 ± 41.4 µg/24 h), significantly decreased by 70.5% as compared to baseline (baseline: 835.3 ± 625.7 nmol/24 h, 303.7 ± 227.5 µg/24 h; *p*_(0–12)_ = 0.016), with a non-significant mean increase of 16.7% as compared to 6-month follow-up [6 months: 239.1 ± 177.3 nmol/24 h, 86.9 ± 64.5 µg/24 h; *p*_(6–12)_ = 0.58]. According to a “per-protocol” methodology analysis, 28.6% (two) patients were FC, 57.1% (four) were PC, and 14.3% (one) patient was UC. On the basis of these evidences, a full or partial control of the disease was observed in 85.7% of cases, corresponding to six of seven patients who reached 12-month follow-up. According to an “intention-to-treat” methodology analysis, 14.3% (two) were FC, 28.6% (four) were PC, and 57.1% (eight) patients were UC. On the basis of these evidences, a full or partial control of the disease was observed in 42.9% of cases, corresponding to six of the 14 patients enrolled in the study and starting treatment. At 12-month follow-up, UFC variably decreased in all the three groups of patients. Indeed, in the two FC patients, a mean UFC reduction of 78.1% (71–85.2%) was observed, whereas a mean UFC reduction of 72.4% (61–91%) and 47.7% was observed in the four PC patients and in the UC patient, respectively.

### Changes in clinical picture

**Fig. 1:**
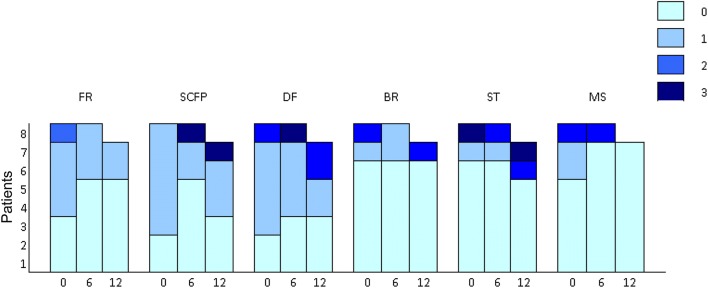
Number of patients with signs of hypercortisolism from baseline (0) to 6 and 12 months of follow-up. FR facial rubor, SCFP supraclavicular fat pad, DF dorsal fat pad, BR bruising, ST striae [0 = no signs; 1 = mild; 2 = moderate; 3 = severe]. MS muscle strength [0 = able to stand easily with arms extended; 1 = able to stand after several efforts without using arms as assistance; 2 = able to stand using arms as assistance; 3 = completely unable to stand]

At months 6 and 12, the majority of patients had an improvement, by at least one category (from severe to moderate; from moderate to mild or from mild to none) or had either the same severity, of signs and symptoms as at baseline (Fig. 1). A relevant improvement was observed in hirsutism: indeed, six (85.7%) of seven women improved FGS. Only one patient (1/7 women, 14.3%) had a worsening of hirsutism. A minority of patients had a worsening of supraclavicular fat pad (1/8; 12.5%), dorsal fat pad (2/8; 25%), bruising (1/8; 12.5%), striae (2/8; 25%), and muscle strength (1/8; 12.5%).

### Changes in metabolic profile

The changes in metabolic parameters from baseline to months 6 and 12 are detailed in Table 3, the changes in the prevalence of metabolic comorbidities are detailed in Table 4, and the changes of different treatments (anti-hypertensive, lipid-lowering, and antidiabetic) are detailed in Table 5.

**Table 3:** Changes in metabolic parameters from baseline to months 6 and 12 (mean ± SD)

Parameter	Baseline^a^ (*M* ± SD)	6 months (*M* ± SD)	*Δ* (0–6)	*p*_(0–6)_	Baseline^b^ (*M* ± SD)	12 months (*M* ± SD)	*Δ* (0–12)	*p*_(0–12)_
Weight (kg)	74.31 ± 16.71	70.10 ± 15.47	− 4.21 ± 5.37	0.05	76.64 ± 16.59	70.5 ± 14.24	− 6.14 ± 8.15	0.047
Body Mass Index (kg/m^2^)	27.66 ± 6.32	26.21 ± 6.62	− 1.44 ± 1.82	0.05	27.97 ± 6.76	25.9 ± 6.98	− 2.07 ± 2.74	0.047
Waist circumference (cm)	100.62 ± 19.8	93.44 ± 13.25	− 7.19 ± 9.39	0.07	102 ± 20.98	90.71 ± 15.14	− 11.28 ± 10.3	0.047
Systolic blood pressure (mmHg)	133.75 ± 23.26	125.25 ± 15.6	− 8.5 ± 18.07	NS	127.14 ± 14.96	130.43 ± 16.96	3.28 ± 8.86	NS
Diastolic blood pressure (mmHg)	82.5 ± 16.03	83 ± 14.31	0.5 ± 18.06	NS	80 ± 15.54	79.43 ± 12.46	− 0.57 ± 9.32	NS
Total cholesterol (mmol/L)	5.25 ± 0.83	5.11 ± 0.93	− 0.14 ± 0.62	NS	5.11 ± 0.79	4.93 ± 1.03	− 0.18 ± 0.78	NS
LDL cholesterol (mmol/L)	2.8 ± 0.71	2.74 ± 0.76	− 0.06 ± 0.59	NS	2.63 ± 0.57	2.66 ± 0.71	0.03 ± 0.71	NS
HDL cholesterol (mmol/L)	2.07 ± 0.46	1.99 ± 0.30	− 0.08 ± 0.34	NS	2.09 ± 0.49	1.96 ± 0.48	− 0.12 ± 0.23	NS
Triglycerides (mmol/L)	2.07 ± 1.06	1.75 ± 0.95	− 0.32 ± 0.52	NS	2.20 ± 1.08	1.53 ± 0.51	− 0.67 ± 0.74	0.07
Fasting plasma glucose (mmol/L)	4.74 ± 0.77	5.25 ± 0.97	0.51 ± 1.12	NS	4.78 ± 0.82	5.17 ± 1.25	0.38 ± 1.18	NS
HbA1c (%)	5.26 ± 0.29	6.65 ± 0.97	1.39 ± 1	0.008	5.3 ± 0.29	6.66 ± 1.28	1.36 ± 1.19	0.031

^a^8 pts

^b^7 pts

**Table 4:** Changes in the prevalence of metabolic comorbidities from baseline to months 6 and 12

Metabolic comorbidities	Baseline	6 months^a^	12 months^b^	*p*
Overweight nr. (%)	3 (37.5)	2 (25)	3 (42.85)	NS
Obesity nr. (%)	2 (25)	2 (25)	1 (14.3)	NS
Visceral obesity nr. (%)	7 (87.5)	7 (87.5)	5 (71.4)	NS
Systemic arterial hypertension nr. (%)	6 (75)	6 (75)	5 (71.4)	NS
Hypercholesterolemia nr. (%)	6 (75)	5 (62.5)	4 (57.1)	NS
Hypertriglyceridemia nr. (%)	5 (62.5)	3 (37.5)	3 (42.85)	NS
IFG and/or IGT nr. (%)	1 (12.5)	4 (50)	2 (28.6)	NS
Diabetes nr. (%)	1 (12.5)	3 (37.5)	3 (42.85)	NS
Metabolic syndrome nr. (%)	4 (50)	3 (37.5)	4 (57.1)	NS

^a^8 pts

^b^7 pts

**Table 5:** Picture of anti-hypertensive, lipid-lowering, and antidiabetic treatments during the study

Hypertension^a^	Baseline (6/8 pts)	6 months (6/8 pts)	Dose variation (0–6)	12 months (5/7 pts)	Dose variation (6–12)
Diet	1	1	1 (=)	0	1(X)
1 drug—nr.	3	3	2 (=), 1 (↑)	4	3 (=), 1 (N)
2 drugs—nr.	0	0	0	1	1 (N)
3 drugs—nr.	1	1	1 (↓)	0	1 (X: 1 DRUG)
≥ 4 drugs—nr.	1	1	1 (=)	0	1DROP OUT

Hypercholesterolemia^b1^	Baseline (6/8 pts)	6 months (5/8 pts)	Dose variation (0–6)	12 months (4/7 pts)	Dose variation (6–12)
Diet	6	5	5 (=), 1 (X)	4	3 (=), 1 (N), 1 (X), 1DROP OUT
+ 1 drug—nr.	1	1	1 (=)	2	1 (=), 1 (N)

Hypertriglyceridemia^b2^	Baseline (5/8 pts)	6 months (3/8 pts)	Dose variation (0–6)	12 months (3/7 pts)	Dose Variation (6–12)
Diet	5	3	3 (=), 2 (X)	3	2 (=), 1 (X), 1 (N)
+ 1 drug—nr.	0	1	1 (N)	1	1 (=)

Type 2 diabetes^c^	Baseline (1/8 pts)	6 months (3/8 pts)	Dose variation (0–6)	12 months (3/7 pts)	Dose variation (6–12)
Diet	1	3	1 (=), 2 (N)	3	2 (=), 1 (N), 1DROP OUT
+ 1 drug—nr.	0	0	0	0	0
+ 2 drugs—nr.	0	3	3 (N)	2	2 (=), 1DROP OUT

(=): patients who did not change the drug dose; (↑): patients who increased the drug dose; (↓): patients who reduced the drug dose; (N): patients who started the treatment; (X): patients who stopped the treatment

^a^Anti-hypertensyve drugs: angiotensin-converting-enzyme inhibitors (ACE-I), angiotensin II receptor blockers (ARBs), diuretics, beta-blockers, calcium antagonists, methyldopa

^b^Lipid-lowering drug: ^1^rosuvastatin, ^2^omega-3 fatty acids

^c^Antidiabetic drugs: metformin, short-acting insulin, long-acting insulin

#### Weight and obesity

Mean BW, BMI, and WC decreased throughout the study. Significant reductions in mean BW and BMI were seen at months 6 and 12. Mean WC showed a trend to a significant reduction at month 6 and was significantly reduced at month 12. At baseline, three (37.5%) had normal weight, three (37.5%) were overweight, and two (25%) patients were obese. At months 6 and 12, respectively, four (50%) and three (42.85%) had normal weight, two (25%) and three (42.85%) were overweight, and two (25%) and one (14.3%) patients were obese. At baseline, visceral obesity was reported in seven (87.5%) patients, confirmed in seven (87.5%) at month 6 and reduced to five (71.4%) at month 12, due to normalization of WC in one FC patient and the drop-out of the UC patient.

#### Blood pressure and hypertension

No significant difference was observed in mean SBP and DBP between baseline, month 6 and month 12. At baseline and month 6, systemic arterial hypertension was observed in six (75%) of eight patients, and, at month 12, in five (71.4%) of seven patients, due to the drop-out of the UC hypertensive patient. At study entry, five of six patients with hypertension were on anti-hypertensive treatment and one was on hyposodic diet; during the study, three patients (one FC and two UC) continued unmodified their therapy, one PC patient reduced the dosage of all three anti-hypertensive drugs, starting reduction after 15 days of pasireotide and then gradually in the following months until stopping one drug, whereas one PC patient increased the dosage of anti-hypertensive drug after 60 days of pasireotide. The PC patient on hyposodic diet therapy at baseline started a new anti-hypertensive drug after 8 months of pasireotide.

#### Lipid profile and dyslipidemia

No significant difference was observed in mean total, LDL, and HDL cholesterol between baseline, month 6 and month 12. Hypercholesterolemia was observed at baseline in six (75%) of eight patients, at month 6 in five (62.5%) of eight patients, due to the normalization of cholesterol in one FC patient, and at month 12 in four (57.1%) of seven patients, due to the normalization of cholesterol in one FC patient, a new diagnosis of hypercholesterolemia in one FC patient, and the drop-out of the UC hypercholesterolemic patient. At baseline, all six patients with a diagnosis of hypercholesterolemia were on diet therapy and one was also in statin therapy. During the study, the entire cohort of patients with hypercolesterolemia continued unmodified their therapy; in the new diagnosed FC patient, diet therapy was added, and only in one UC patient, a statin therapy was added after 10 months of treatment.

No significant difference was observed in mean triglycerides at month 6, but a trend to a significant reduction was observed at month 12. Hypertriglyceridemia was observed at baseline in five (62.5%) of eight patients, at month 6 in three (37.5%) of eight patients, due to the normalization of triglycerides in two PC patients, and at month 12 in three (42.85%) of seven patients, due to the normalization of triglycerides in one UC patient and a new diagnosis of hypertriglyceridemia in one PC patient. At baseline, all five patients with a diagnosis of hypertriglyceridemia were on diet therapy. During the study, the entire cohort of patients with hypertriglyceridemia continued unmodified their therapy; in one FC patient, a supplementation with omega-3 fatty acids was added after 4 months of treatment, and in the new diagnosed PC patient diet therapy was added during the last 6 months of treatment.

#### Glucose metabolism

No significant difference was observed in mean FPG both at months 6 and 12. Particularly, although FPG increased by 85.7% during the first 2 months of treatment, this transient increase did not reach statistical significance (baseline: 4.7 ± 0.8 mmol/L; 2 months: 8.4 ± 7.6 mmol/L; *p*_0–2_ = 0.375); in fact, during the following 4 months of treatment, until 6-month follow-up, FPG non-significantly decreased by 14.9% (2 months: 8.4 ± 7.6 mmol/L; 6 months: 5.2 ± 1 mmol/L, *p*_2–6_ = 0.297), non-significantly re-increasing by 4.7% (12 months: 5.2 ± 1.2 mmol/L; *p*_6–12_ = 0.938) during the last 6 months of treatment in the remaining seven patients, until 12-month follow-up.

Mean HbA1c was significantly increased both at months 6 and 12, compared to baseline. Particularly, HbA1c significantly increased by 23% during the first 2 months of treatment, remaining, however, within target value ≤ 7.5% (baseline: 5.3 ± 0.3%; 2 months: 6.5 ± 1%; *p*_0–2_ = 0.008). Nevertheless, during the following 4 months of treatment, until 6-month follow-up, HbA1c remained nearly stable, with a non-significant slight increase of 3% (2 months: 6.5 ± 1%; 6 months: 6.6 ± 1%, *p*_2–6_ = 0.547), followed by a further non-significant slight increase of 1.8% registered during the last 6 months of treatment in the remaining seven patients, until 12-month follow-up (12 months: 6.7 ± 1.3%; *p*_6–12_ = 0.563).

An increase in mean FPG and HbA1c was observed during the first 2 months of treatment, after which FPG and HbA1c generally plateaued or even normalized, although often requiring specific antidiabetic treatments. In particular, after the first 2 months of treatment and considering the seven patients who completed the 12-month follow-up, FPG remained normal in three (42.8%), normalized in two (28.6%) cases, improved without normalization in one (14.3%), and deteriorated only in one (14.3%) case. HbA1c remained normal in two (28.6%), stably impaired within target value ≤ 7.5% in three (42.8%) and exceeding 7.5% in two (28.6%) cases. The UC patient who withdrew the consent for poor compliance and discontinued the treatment at 6-month follow-up, showed increases in FPG during the first 2 months of treatment, after which levels normalized with specific antidiabetic treatments, whereas HbA1c progressively increased, but remained within target value ≤ 7.5% until the treatment withdrawal. A prediabetes, including IFG and/or IGT, was observed at baseline in one (12.5%) of eight patients, at month 6 in four (50%) of eight patients, due to a new diagnosis of prediabetes in three (two PC and one UC) patients, and at month 12 in two (28.6%) of seven patients, due to a new diagnosis of diabetes in the FC patient with prediabetes at baseline and the normalization of glucose metabolism in one PC patient. Diabetes was observed at baseline in one (12.5%) of eight patients, at month 6 in three (37.5%) of eight patients, due to a new diagnosis of diabetes in two (one PC and one UC) patients, and at month 12 in three (42.85%) of seven patients, due to a new diagnosis of diabetes in the FC patient with prediabetes at baseline and the drop-out of the UC diabetic patient. Summarizing, during pasireotide treatment, the patient (12.5%) with diabetes remained diabetic, five (62.5%) of eight patients worsened their glucose metabolism (two from normal to diabetes, two from normal to prediabetes, and one from prediabetes to diabetes), one (12.5%) patient, after an initial worsening (from normal to prediabetes), normalized glucose metabolism, and the remaining patient (12.5%) with normal glucose metabolism at study entry remained stably normal.

**Fig. 2:**
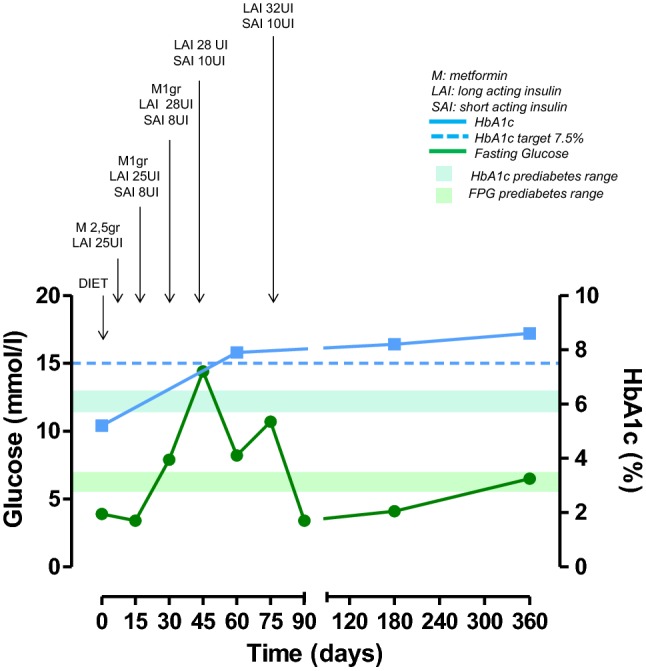
Changes in glucose (mmol/L) and HbA1c (%) in the patient with diabetes at baseline, treated with pasireotide at least 6 months

**Fig. 3:**
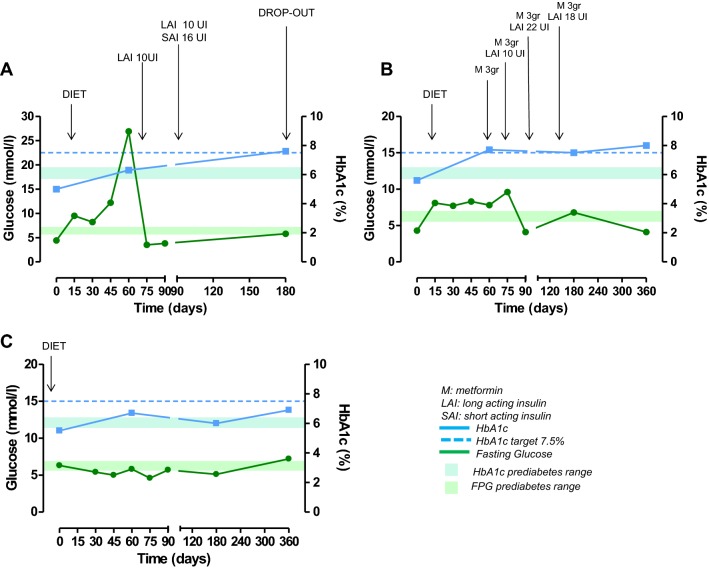
Changes in glucose (mmol/L) and HbA1c (%) in the three patients treated with pasireotide at least 6 months who received a new diagnosis of diabetes. **a**, **b** Normal glucose metabolism at baseline; **c** prediabetes at baseline

**Fig. 4:**
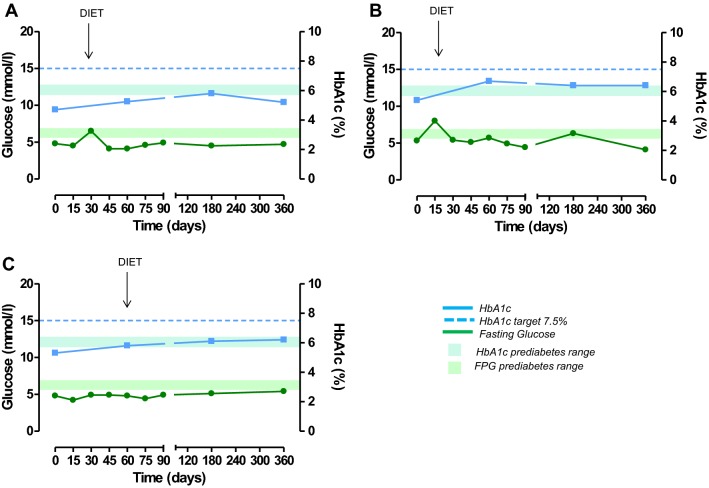
Changes in glucose (mmol/L) and HbA1c (%) in the three patients with normal glucose metabolism at baseline who received a new diagnosis of prediabetes

At study entry, the only patient with a diagnosis of diabetes was on mediterranean dietary regimen; during the study, metformin and long-acting insulin were added after 8 days and short-acting insulin after 15 days of treatment; after 45 days, metformin was withdrawn, keeping on with short-acting/long-acting insulin (Fig. 2). In two patients with normal glucose metabolism at study entry, becoming diabetic, new antidiabetic therapies were added during the study. In one UC patient, a mediterranean dietary regimen was suggested after 15 days, long-acting insulin after 75 days and short-acting insulin after 90 days of treatment (Fig. 3a); in this patient, metformin was not prescribed due to renal failure. In one PC patient, a mediterranean dietary regimen was suggested after 15 days, metformin after 60 days, and long-acting insulin after 75 days of treatment (Fig. 3b). In the FC patient (12.5%) with prediabetes who developed diabetes after 12 months of treatment, the mediterranean dietary regimen was continued during the study (Fig. 3c). The three patients who received a new diagnosis of prediabetes, were treated according to a mediterranean dietary regimen and strictly monitored on the basis of daily self-monitoring of blood glucose by home finger stick, FPG and HbA1c. A complete normalization of glucose metabolism was obtained in one PC patient (Fig. 4a) and an improvement or stabilization in the other two patients (one PC and one UC) (Fig. 4b, c).

The data included in this section refer to patients enrolled in the study and reached at least 6–12 months of treatment, excluding the six patients dropped out from the study before reaching the 6-month follow-up. The safety section includes data on glucose metabolism in the entire cohort of 14 patients enrolled in the study.

#### Metabolic syndrome

A clear diagnosis of MS was performed at baseline in four (50%) of eight patients, at month 6 in three (37.5%) of eight patients, due to the normalization of FPG in one FC patient and triglycerides in one PC patient, and a new diagnosis of MS in one UC patient, and at month 12 in four (57.1%) of seven patients, due to a new diagnosis in two patients, related to the increase in FPG in one FC patient and triglycerides in one PC patient, and the drop-out of the UC patient suffering from MS.

#### Clinical picture and metabolic profile: relation with UFC changes

In the entire series of clinical signs and symptoms as well as in the entire series of metabolic parameters, no significant differences were observed between FC, PC, and UC patients, both at 6 and 12 months.

### Safety

In the 14 patients enrolled in the current study, at baseline, seven (50%) presented a normal glucose metabolism, four (28.6%) had prediabetes, and three (21.4%) patients had diabetes.

Among the seven patients with a normal glucose metabolism at study entry, five (71.4%) reported new hyperglycemia-related AEs; in particular, new diagnosis of prediabetes occurred in three (42.8%) and new diagnosis of diabetes in two (28.6%) cases. The remaining two (28.6%) patients with a normal glucose metabolism at study entry maintained the glucose state during treatment, for 15 days until pasireotide discontinuation in one case, and for the entire period of 12 months in the remaining case.

Among the seven patients with impaired glucose metabolism at study entry, five (71.4%), including two of the four patients with prediabetes and the three patients with diabetes, deteriorated glucose metabolism during treatment; in particular, a worsening of prediabetes or diabetes occurred in four (57.1%) cases and new diagnosis of diabetes in one (14.3%) case. The remaining two (28.6%) patients with prediabetes at study entry dropped out from the study after 7 days of treatment, without intermediate controls. No patients dropped out from the study for hyperglycemia-related AEs.

Summarizing, during treatment, six (42.8%) patients maintained (three, 21.4%) or developed (three, 21.4%) prediabetes, six (42.8%) patients maintained (three, 21.4%) or developed (three, 21.4%) diabetes, and two (14.4%) patients maintained normal glucose metabolism.

Three (21.4%) patients, including two with normal glucose metabolism and one with prediabetes at baseline, received a new diagnosis of diabetes. In all three patients, a mediterranean dietary regimen was suggested as the first-line approach, with strict monitoring of FPG, HbA1c, and daily self-monitoring of blood glucose by home finger stick. Despite this first-line treatment, in one of the two patients with normal glucose metabolism at baseline, to manage the increase in FPG, HbA1c and blood glucose by home finger stick, a second-line approach was required after 75 days of treatment: metformin was not considered due to renal failure, preferring long-acting insulin, which induced normalization of blood glucose in 7 days. However, the addition of a short-acting insulin was required after 90 days of treatment to maintain HbA1c in the target value (≤ 7.5%) (Fig. 3a). In the remaining patient with normal glucose metabolism at baseline, to manage the increase in FPG and HbA1c, metformin therapy was added, after 60 days of treatment, as second-line approach; however, the addition of long-acting insulin after 75 days of treatment, and dose increase after 90 days of treatment, was required, as third-line approach, to normalize FPG, and to maintain HbA1c in the target value (Fig. 3b). In the patient with prediabetes at baseline, who developed diabetes during treatment, the first-line dietary regimen was sufficient to control FPG and maintain HbA1c in the target value (Fig. 3c).

Three (21.4%) patients, displaying a normal glucose metabolism at baseline, received a new diagnosis of prediabetes. In all three patients, a mediterranean dietary regimen was suggested as first-line approach, with strict monitoring of FPG, HbA1c, and daily self-monitoring of blood glucose by home finger stick. A complete normalization of FPG and HbA1c was obtained in one case (Fig. 4a) and an improvement or stabilization in the other two cases (Fig. 4b, c).

**Fig. 5:**
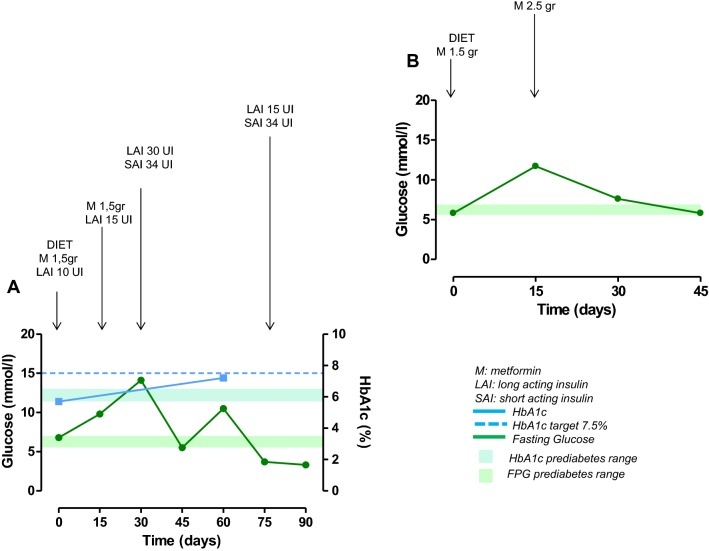
Changes in glucose (mmol/L) and HbA1c (%) in the two patients with diabetes at baseline, treated with pasireotide for 90 (**a**) and 45 days (**b**), respectively

Three (21.4%) patients, displaying a previous diagnosis of diabetes, experienced a worsening in glucose profile. One patient already at baseline in mediterranean dietary regimen, after 8 days of treatment, on the basis of further increased glucose self-monitored by home finger stick, required the addition of a first-line combined treatment with metformin plus long-acting insulin. Despite this first-line combined approach, to manage these relevant increases in blood glucose, after 15 days of treatment, the metformin dose was reduced, and short-acting insulin added. However, considering that this second-line approach was not sufficient to control FPG, after 30 days of treatment, an increase in long-acting insulin dose was performed, and 15 days after, metformin treatment was interrupted, and short-acting insulin dose increased. Lastly, considering that this third-line approach was still not sufficient to control FPG and to reduce HbA1c, a further increase in long-acting insulin dose was required after 75 days of treatment, with normalization of FPG, but persistence of HbA1c out of target value (Fig. 2). The second diabetic patient at baseline was already in mediterranean dietary regimen, together with metformin plus long-acting insulin. Despite this treatment, to manage the increase in FPG, after 15 days of treatment, an increase in long-acting insulin dose was required. However, considering that this first-line approach was not sufficient to control FPG, 15 days after, the metformin treatment was interrupted, long-acting insulin dose increased, and short-acting insulin added, with consequent normalization of FPG and maintenance of HbA1c in the target value (Fig. 5a). The third diabetic patient at baseline was already in mediterranean dietary regimen and metformin. Despite this treatment, to manage the increase in FPG, after 15 days of treatment, an increase in metformin dose was required to normalize FPG. No HbA1c intermediate controls were available due to early drop-out from the study after 45 days of treatment (Fig. 5b).

Lastly, a mediterranean dietary regimen and a strict daily self-monitoring of blood glucose by home finger stick were suggested to the remaining three (21.4%) patients with prediabetes at baseline, until their drop-out from the study after 7 days in two cases and 45 days in the other one.

Less commonly reported AEs are herein described and are detailed in Table 6.

**Table 6:** Prevalence of less common reported adverse events stratified according to the degree

Adverse event	N° pts (%)	Mild	Moderate	Severe	Very severe	Action taken
Fatigue and asthenia	4 (28.6)	1	1	1	1	SP
Nausea	3 (21.4)	2	0	1^a^	0	SP; Metoclopramide/ondansetron^a^
Vomiting	2 (14.3)	0	0	1	1^a^	SP; Metoclopramide/ondansetron^a^
Abdominal pain	2 (14.3)	2	0	0	0	SP
Diarrhoea	2 (14.3)	0	0	1^a^	1^a^	Loperamide
Transient GGT elevation	2 (14.3)	1	1	0	0	SP
Transient ALT elevation	1 (7.1)	0	1	0	0	SP
Gallstones	1 (7.1)	1^a^	0	0	0	Ursodeoxycholic acid
Biliary sludge	1 (7.1)	1^a^	0	0	0	Ursodeoxycholic acid
QTc elevation	1 (7.1)	1	0	0	0	SP
Hypocortisolism	1 (7.1)	0	1^a^	0	0	Reduction of pasireotide dose
Headache	1 (7.1)	1	0	0	0	SP
Anemia	1 (7.1)	1^a^	0	0	0	Iron supplementation
Anxiety	1 (7.1)	1^a^	0	0	0	Benzodiazepines
Peripheral oedema	1 (7.1)	1^a^	0	0	0	Anti-hypertensive drug change

*GGT* glutamyltransferase, *ALT* alanine aminotransferase, *QTc* corrected QT interval, *SP* spontaneously resolved

^a^Not spontaneously resolved, requiring specific interventions

Fatigue and asthenia, from mild to very severe, were reported in four (28.6%) patients, spontaneously resolved without pasireotide discontinuation in 15–30 days. During the study, nausea occurred in three (21.4%) patients, whereas vomiting and abdominal pain in two (14.3%) patients. Nausea, vomiting, and abdominal pain were spontaneously resolved in all cases in 15–60 days, except in one with severe nausea and very severe vomiting, who was treated with the dopamine receptor antagonist metoclopramide, as first-line approach, and, due to the persistent worsening of the symptoms, with the serotonin 5-HT3 receptor antagonist ondansetron, as second-line approach; after 45 days of treatment, the patient, because of the persistent worsening of these AEs, withdrew the consent. Two (14.3%) patients reported severe to very severe diarrhoea, managed with the μ-opioid receptor agonist loperamide, as first-line approach; however, despite the reduction in number of episodes, the persistence of diarrhoea induced the patients to withdraw the consent and discontinue treatment after 15 and 45 days, respectively. Mild to moderate transient elevations in glutamyl transferase (GGT) and alanine aminotransferase (ALT) (< 3 times ULN) were reported in two (14.3%) patients and one (7.1%) patient, respectively, spontaneously resolved without pasireotide discontinuation in 1–5 months. One (7.1%) patient with a normal gallbladder on ultrasonographic examination at baseline, during treatment displayed two millimetric gallstones (≤ 4 mm) and detectable biliary sludge, managed with ursodeoxycholic acid, which induced resolution of lithiasis and biliary sludge. One (7.1%) patient had a newly occurring mild prolongation of the corrected QT interval of more than 480 ms, but less than 500 ms, not requiring treatment interruption and spontaneously resolved in 60 days. A moderate hypocortisolism-related AE was reported in one (7.1%) patient, resolved with a reduction in the pasireotide dose. Lastly, less frequently pasireotide associated AEs, reported each in one (7.1%) patient, were: transient and occasional mild headache, spontaneously resolved in 45 days; transient mild anemia, treated for 3 months with oral iron supplementation, until normalization of haemoglobin; mild anxious syndrome, treated and resolved with low daily doses of benzodiazepines; and mild peripheral oedema, related to amlodipine treatment, resolved after replacement of amlodipine with lisinopril. No deaths or hospitalizations were registered during treatment.

## Discussion

The current study, performed in a limited cohort of patients with de novo or persistent CD, demonstrated that pasireotide, at a median dose of 900 µg bid sc, induced a full disease control in 37.5% and 28.6% of patients, according to a “per-protocol” methodology analysis, and in 21.4% and 14.3% of patients, according to an “intention-to-treat” methodology analysis, after 6 and 12 months of treatment, respectively. It is noteworthy that full or partial disease control was obtained in 75% of patients after 6 months and in 85.7% after 12 months of treatment according to the “per-protocol” methodology analysis, whereas in 42.9% of patients, both at 6 and 12 months of treatment, according to the “intention-to-treat” methodology analysis. The discrepancy between the success rates obtained with the two methodological analyses highlighted the difference that may be obtained in the evaluation of a treatment response.

The efficacy of pasireotide treatment was firstly evaluated in a multicenter open-label phase II study in patients with de novo or persistent/recurrent CD, at the dose of 600 µg bid for 15 days, displaying UFC decrease in 76%, and UFC normalization in 17% of cases [21]. After 6 months of the extension period, 56% of patients entering the extension phase had lower UFC than at baseline, and 22% had normal UFC [22]. However, the largest experience on pasireotide efficacy derives from the phase III study in 162 CD patients, where pasireotide treatment induced, after 6 months, full disease control in 14.6% (600 μg bid) and 26.3% (900 μg bid) of patients without drug dose up-titration, and in 15.9% (600 μg bid) and 28.8% (900 μg bid) of patients, regardless of dose increase. After 12 months of treatment, UFC normalization was maintained in 13.4% (600 μg bid) and 25% (900 μg bid) of patients. Regardless of dose increase, full or partial disease control was obtained in 34.2% (600 μg bid) and 41.3% (900 μg bid) of patients after 6 months, and in 29.3% (600 μg) and 27.5% (900 μg) of patients after 12 months [10]. More recently, pasireotide was reported to control UFC levels over 24 months of treatment in 20 (34.5%) of 58 patients who entered the extension phase III study [12], and over 60 months in 11 (68.8%) of 16 patients who received 5 years of pasireotide treatment [23].

Notably, in these large experiences, UFC reduction was accompanied by improvements in symptoms and signs as well as in metabolic profile and comorbidities of CD, also maintained during long-term treatment [10–12, 23]. Facial rubor, supraclavicular and dorsal fat pads, and bruising improved, whereas BW, BMI, WC, blood pressure, total and LDL cholesterol were significantly reduced, as well as depression and QoL scores improved [10–12]. Surprisingly, the majority of these improvements were not limited to patients in whom a normal UFC level was achieved, being present even in case of partial disease control, suggesting that clinical beneficial effects may be obtained also in patients who did not normalize, but experienced a significant reduction in cortisol secretion [11, 12].

The current study appears to confirm the majority of the findings reported in the phase III study. In fact, in the eight patients who reached at least 6-month follow-up, a relevant improvement in the typical clinical signs and symptoms characterizing hypercortisolism was reported, particularly in facial rubor, supraclavicular fat pad, bruising, hirsutism, and muscle strength, associated with a significant improvement in BW, BMI, WC, and a slight non-significant reduction in the prevalence of visceral obesity, hypercholesterolemia, and hypertriglyceridemia. In line with the previous evidence, the observed improvements were similar in FC, PC, and UC patients, confirming that pasireotide effects on clinical picture and metabolic profile were present also in patients with cortisol reduction, although mostly evident in patients with complete normalization of cortisol secretion. Interestingly, these results seem to confirm not only data of the phase III study, but also data reported in different studies, mainly small series or case reports [23–30], published during the last years, that have documented improvements in signs and symptoms [23–28], BW [22, 23, 26–29], WC [26–29], blood pressure [22, 23, 25–30], and lipid profile [28].

Therefore, the current study, although conducted in a limited series of patients, confirmed that pasireotide treatment is able to induce full or partial hormone control in a majority of CD patients treated for 6–12 months, associated with a beneficial effect on clinical picture and main comorbidities, reinforcing that clinical benefit may be obtained also in patients who experienced a significant reduction without cortisol normalization.

In the current study, during the entire period of treatment, in the 14 patients evaluated for safety profile, hyperglycemia-related AEs were the most commonly reported AE, occurring in 71.4% of patients, including 35.7% with a new diagnosis of hyperglycemia-related AEs and 35.7% with a worsening of their basal impairment of glucose metabolism; noteworthy, 14.3% of patients with prediabetes at baseline dropped out from the study after 7 days of pasireotide treatment without intermediate controls, and 14.3% of patients maintained their normal glucose metabolism during the study. This safety profile evidence appears similar to those previously reported [10, 12, 23–29]. Particularly, in the largest phase III clinical trial experience, hyperglycemia-related AEs were observed in 72.8% of patients at 12-month follow-up [10], in 79.1% during long-term treatment up to 24 months [12] and in 93.8% up to 60 months [23]. However, in the current experience, no patients dropped out from the study due to hyperglycemia-related AEs and all cases were safely managed. Conversely, in the phase III study, severe and very severe hyperglycemia-related AEs induced treatment discontinuation in 5.6% of patients [10].

To our knowledge, despite some suggestions have been provided, the best way to manage pasireotide-related AEs is still a matter of debate. The current study aimed at detailing this peculiar aspect, describing the approach followed in one of the major centers with a depth and long experience in the CD management, involved in the phase III trial on pasireotide treatment, offering a view on “safety management” in a tertiary care center.

In the current study, hyperglycemia risk was strictly monitored, more closely if an impaired glucose metabolism was evident at study entry. In case of a new diagnosis of diabetes, a mediterranean dietary regimen was suggested as first-line approach, an addition of metformin therapy and/or long-acting insulin was preferred as second-line approach, whereas the addition of a short-acting insulin was selected as third-line approach. In case of diabetes treated with metformin *plus* long-acting insulin at study entry, metformin and long-acting insulin dose adjustment was preferred as first-line approach, whereas a reduction, until interruption, of metformin doses and the addition of a short-acting insulin was selected as second-line approach. Lastly, in case of a new diagnosis of prediabetes or prediabetes present at baseline, a mediterranean dietary regimen and a strict monitoring were suggested as preferred approach. In line with the previous findings, also in the current experience, in patients treated with pasireotide for at least 6 months, both FPG and HbA1c increased soon after the initiation of pasireotide treatment. After the first 2 months, concomitantly with the initiation of a dietary regimen and/or antidiabetic treatment, FPG remained normal in 42.8% of patients, and normalized in 28.6% of patients who reached 12 months of treatment, whereas HbA1c remained normal in 28.6%, stably impaired within target values of 7.5% in 42.8%, and exceeding target values of 7.5% in 28.6% of patients, therefore, testifying a good control in 71.4% of patients. However, this safety management experience was conducted before the significant progress in understanding the fundamental pathophysiology of hyperglycemia secondary to pasireotide treatment, not considering the “glucagon-like peptide (GLP-1) pathway”, which appeared later in healthy volunteers to be involved as main mechanism of pasireotide-induced hyperglycemia [14, 15, 31–35]. Indeed, only after study completion, a panel of European experts in pituitary disease and diabetes suggested “expert recommendations” to manage hyperglycemia in CD patients treated with pasireotide [15].

Experts recommended that after lifestyle changes and medical treatment with metformin, if the control of glucose metabolism is not achieved or maintained [HbA1c level of > 7.0–7.5% (> 53–58 mmol/mol)], combination therapy with agents targeting the incretin pathway is recommended [15, 31]. First, therapy with metformin and dipeptidyl peptidase-4 (DPP-4) inhibitor may be established [15]. If glycemic target values are not reached, DPP-4 inhibitor may be replaced by a GLP-1 receptor agonist [15], with the advantages of higher HbA1c-lowering effect, without increasing the risk of hypoglycemia, and with the potential ability to reduce BW. If hyperglycemia remains uncontrolled by these combinations, establishing insulin therapy together with maintained metformin treatment may be necessary [15]. In these cases, initial combination therapy of metformin with long-acting basal insulin, targeting FPG, may be the first option. If the individual HbA1c target values are not met or the postprandial glucose values are high with basal insulin, short-acting prandial insulin therapy has to be finally established [15]. If uncontrol of glucose metabolism persists, pasireotide dose reduction or discontinuation should be considered, and also after pasireotide discontinuation, glucose monitoring should be performed according to clinical practice [15, 34]. However, the efficacy of different treatment regimens needs to be further explored in clinical trials in CD patients. Moreover, further research on the mechanism underlying the effects of pasireotide on insulin synthesis and secretion, and insulin sensitivity, in CD patients will help to develop specific guidelines for managing pasireotide-induced hyperglycemia in the future. It is conceivable that the control of hyperglycemia obtained in the current experience should have been better achieved and maintained by treating the underlying specific causes of pasireotide-induced hyperglycemia, and applying the subsequently established recommendations, therefore, limiting insulin therapy to a selected number of cases with persistent uncontrolled hyperglycemia, despite treatment with metformin, DPP-4 inhibitor, and GLP-1 receptor agonist. Probably, nowadays, acting on GLP-1 pathway, HbA1c target value could also be more rapidly and/or more stably achieved and maintained.

Less commonly reported AEs, in line with previous evidence, included gastrointestinal disturbances, cholelithiasis, transient elevations of liver enzymes, QT prolongation and hypocortisolism-related AEs [10, 12, 23, 25, 27–30]. Gastrointestinal disorders, mainly nausea, vomiting, abdominal pain and diarrhoea, are the most common AEs, for which a strict monitoring during pasireotide treatment is recommended, maintaining a “wait and see” approach [34, 35]. Indeed, they usually occur at the beginning of treatment and either disappear or become easily tolerable within the first months of treatment. In selected cases with persistent and/or worsening AEs, symptomatic drugs or pasireotide treatment reduction or discontinuation are necessary. In the current study, in the majority of cases, these AEs spontaneously resolved in 2 months; however, some patients had persistent or worsening AEs requiring intervention. In particular, one (7.1%) patient with severe nausea and very severe vomiting was treated with metoclopramide, as first-line approach, and, due to the persistent and worsening symptoms, with ondansetron as second-line approach. However, after 45 days of pasireotide treatment, the patient withdrew the consent, due to the persistence of these AEs. In the two (14.3%) patients reporting severe to very severe diarrhoea, loperamide treatment was the first approach, with reduction of the daily episodes of diarrhoea. However, after 15 and 45 days of pasireotide treatment, respectively, the two patients withdrew the consent, due to the persistence of the AE. As far as liver and gallbladder function was concerned, a careful monitoring is recommended, and treatment should be discontinued if the patient develops jaundice, clinical signs of significant liver dysfunctions, such as severe GGT elevations, sustained transaminase increase (five times the ULN or greater), or transaminase increase (three times ULN) associated with concurrent bilirubin elevation (greater than two times ULN) [34, 35]. However, in the majority of cases, liver enzyme elevations returned to normal spontaneously, without pasireotide discontinuation [34], such as in the three (21.4%) patients described in the current study, where only one (7.1%) patient, reporting gallstones and detectable sludge, required ursodeoxycholic acid treatment to obtain lithiasis and biliary sludge resolution.

Moreover, in consideration of the possible action of pasireotide on QT interval, caution needs to be done also in patients who have congestive heart failure (NYHA Class III or IV), unstable angina, sustained ventricular tachycardia, clinically significant bradycardia, advanced heart block, history of acute myocardial infarction or in patients with risk factors for *torsade de pointes* as patients with hypokalemia, congenital long QT prolongation or family history of long QT syndrome, or in patients treated with concomitant medications known to prolong QT interval [35]. Increase of QTcF > 480 ms should trigger a referral to a cardiologist, with discontinuation of pasireotide in those patients with confirmed QTcF > 500 ms [34]. Hypokalemia or hypomagnesemia must be corrected prior to initiating therapy and monitored thereafter and an electrocardiography is recommended before, 1 week after the beginning of the treatment, and thereafter periodically [34]. One (7.1%) patient in the current study had a newly occurring prolongation of the QTc interval of more than 480 ms, but less than 500 ms, therefore, considered mild, not requiring pasireotide treatment interruption and spontaneously resolved in 2 months.

Lastly, pasireotide treatment may lead, according to its pharmacological activity, to a rapid and marked decrease in serum cortisol, potentially inducing hypocortisolism; therefore, patients need to be advised, monitored, and instructed on how to manage signs and symptoms of hypocortisolism as weakness, fatigue, anorexia, nausea, vomiting, and hypotension [34, 35]. If necessary, pasireotide dose reduction or interruption and/or a temporary exogenous steroid replacement therapy should be evaluated [34, 35], such as in the current study occurred in one (7.1%) patient, in whom hypocortisolism was resolved with a reduction of the pasireotide dose. Certainly, nowadays, the most common AEs related to pasireotide treatment are better known and managed in the daily clinical practice, less frequently limiting the drug use.

In conclusion, the current study performed in a limited cohort of patients with de novo or persistent CD demonstrated that pasireotide treatment is able to induce control of cortisol secretion in 75% of patients treated for at least 6 months and in 85.7% of patients treated for at least 12 months, with full disease control in 37.5% and 28.6% after 6 and 12 months, respectively. A beneficial effect was observed on the clinical picture, with a significant improvement in BW, BMI, WC, and a slight non-significant reduction in the prevalence of visceral obesity, hypercholesterolemia, and hypertriglyceridemia, independently from pasireotide effects on UFC normalization. Although the expected deterioration of glucose metabolism represented the most commonly reported AE, occurring in more than 70% of cases, and requiring specific antidiabetic treatments, in the current experience no patients dropped out from the study due to hyperglycaemia and all cases were safely and successfully managed. A stepwise approach using a dietary regimen, metformin, long-acting, and short-acting insulin was able to obtain, after an initial impairment in HbA1c, a stabilization within target value in the majority of patients, exceeding the suggested target value in a minority of cases. Additional AEs of interest were gastrointestinal disorders, spontaneously resolved in the majority of cases, except in three patients treated with symptomatic drugs and the rare occurrence of millimetric gallstones and biliary sludge, successfully resolved with appropriate medical treatment, whereas the rare occurrence of hypocortisolism-related AEs was resolved with a reduction in the pasireotide dose. The current study, reporting the experience of one of the major centers involved in the phase III trial on pasireotide efficacy in a limited series of patients with CD, confirmed that pasireotide may be considered a valid and safe option for CD patients either de novo for pituitary surgery or experiencing a failure of pituitary surgery.
